# Description of a Novel Adhesin of *Mycobacterium avium* Subsp. *paratuberculosis*

**DOI:** 10.1155/2014/729618

**Published:** 2014-07-22

**Authors:** Mariana Noelia Viale, Gabriela Echeverria-Valencia, Pablo Romasanta, María Laura Mon, Marisa Fernandez, Emilio Malchiodi, María Isabel Romano, Andrea Karina Gioffré, María de la Paz Santangelo

**Affiliations:** ^1^Instituto de Biotecnología, Instituto Nacional de Tecnología Agropecuaria, 1686 Hurlingham, Buenos Aires, Argentina; ^2^Instituto de Estudios de la Inmunidad Humoral (IDEHU), Facultad de Farmacia y Bioquímica, Universidad de Buenos Aires, 1113 Ciudad Autónoma de Buenos Aires, Argentina

## Abstract

The binding and ingestion of* Mycobacterium avium* subsp. *paratuberculosis* (MAP) by host cells are fibronectin (FN) dependent. In several species of mycobacteria, a specific family of proteins allows the attachment and internalization of these bacteria by epithelial cells through interaction with FN. Thus, the identification of adhesion molecules is essential to understand the pathogenesis of MAP. The aim of this study was to identify and characterize FN binding cell wall proteins of MAP. We searched for conserved adhesins within a large panel of surface immunogenic proteins of MAP and investigated a possible interaction with FN. For this purpose, a cell wall protein fraction was obtained and resolved by 2D electrophoresis. The immunoreactive spots were identified by MALDI-TOF MS and a homology search was performed. We selected elongation factor Tu (EF-Tu) as candidate for further studies. We demonstrated the FN-binding capability of EF-Tu using a ligand blot assay and also confirmed the interaction with FN in a dose-dependent manner by ELISA. The dissociation constant of EF-Tu was determined by surface plasmon resonance and displayed values within the *μ*M range. These data support the hypothesis that this protein could be involved in the interaction of MAP with epithelial cells through FN binding.

## 1. Introduction

Paratuberculosis (PTB) is a chronic granulomatous enteritis of domestic and wild ruminants. This disease involves extensive mycobacterial shedding, which accounts for the high contagiousness, and ends with fatal enteritis. Decreases in weight, milk production, and fertility produce severe economic loss [1]. The etiological agent of PTB is* Mycobacterium avium* subsp.* paratuberculosis* (MAP). MAP enters the intestinal tissue through M cells present in the dome epithelium covering the continuous Peyer's patches in the distal ileum [2, 3]. Initially, the pathogen interacts with proteins of the extracellular matrix (ECM), which function as ligands for bacterial adhesion. Fibronectin (FN) binding is required for attachment and internalization of MAP by the epithelial cells and *β*1 integrins have been identified as the host cell receptors for FN-opsonized mycobacteria* in vitro* and* in vivo* [4]. M cells have the distinctive characteristic of displaying *β*1 integrins on their luminal face at high density; therefore, the presence of these integrins on M cells may explain why these cells are the entry of the bacteria. The interaction between MAP and FN is explained by the presence of the FN-binding proteins called adhesins. Different adhesins from several pathogens were identified as virulence factors [5–9]. Adhesins may also induce strong protective immunity in the host and, thus, remain attractive vaccine targets. For instance a 27-kDa outer membrane protein from* Salmonella typhi* binds to laminin and induces a strong protective antibody response in animal models and humans [10]. In addition, the antigen 85 complex (Ag85) was the first family of mycobacterial proteins to be identified as having FN-binding capability. Members of the Ag85 complex were described as a mycobacterial adhesins firstly in* M. tuberculosis* [11] and then in several mycobacterial species [12–15]. The members of this complex are found within the outer envelope and culture supernatants of mycobacteria and are immunodominant antigens [16, 17]. Furthermore, these proteins possess mycolyltransferase activity and catalyze the synthesis of the most abundant glycolipid of the mycobacterial cell wall, trehalose 6,6-dimycolate (TDM) [18]. Another important adhesin described in mycobacteria is the fibronectin attachment protein (FAP). FAP is a member of a family of FN-binding proteins present in several species of mycobacteria that mediate the attachment and internalization of these bacteria by epithelial cells* in vitro* [19–23]. This protein is also called APA (for alanine-proline-rich antigen) and is encoded by a gene annotated as MAP1569 in the MAP K-10 strain. Although the MAP-APA is not an immunodominant antigen, it activates dendritic cells and induces a Th1 polarization [24]. Furthermore, MAP-infected cattle showed a strong humoral response to recombinant APA assayed by Western blot and ELISA [7]. In a previous study conducted by our group, APA was detected mainly in the culture supernatant filtrates, demonstrating that this protein is predominantly secreted [7]. Other cell wall proteins thus could interact with FN to facilitate complex formation and, in this way, allow adherence to epithelial cells.

With all this in mind, we hypothesized that molecules with similar structure, even those from nonrelated microorganisms, could have conserved adhesin functions. In the present study, we searched for conserved adhesins within a large panel of surface immunogenic proteins of MAP and investigated a possible interaction with FN. By using the ligand blot assay (LBA), we confirmed the binding properties of a protein previously described in other bacteria and identified a novel surface component with FN-binding activity in MAP. The protein-protein interactions revealed by LBA were confirmed by ELISA binding assays and surface plasmon resonance (SPR) in order to determine the dissociation constant (KD).

## 2. Materials and Methods

### 2.1. Bacterial Strains and Culture Media

All cloning steps were performed in* Escherichia coli* DH5*α*.* E. coli* BL21(*k*DE3) was used for recombinant protein production.* E. coli* was grown in Luria Bertani (LB) broth or on LB agar. When necessary, ampicillin was added to the medium at a concentration of 100 *μ*g/mL. MAP was grown in Middlebrook 7H9 medium (Difco Laboratories, USA), 0.05% Tween 80, 0.5% glycerol, AD (0.5% bovine serum albumin, 0.2% glucose), and mycobactin (2 *μ*g/mL).

### 2.2. Preparation of Cell Wall Protein Fraction of MAP

MAP cultures were harvested at midlog phase, centrifuged at 14,000 ×g for 20 min at room temperature and washed twice with phosphate buffered saline (PBS). Cell pellets were resuspended in lysis buffer (PBS, 1 mM EDTA) with 1 mM phenylmethanesulfonyl fluoride (PMSF), an inhibitor of serine proteases, and then this suspension was probe sonicated in an ice bath for 15 min with pulses of 1 min on, 1 min off in a Branson Sonifier S250. Cell wall proteins were obtained as previously described by Hirschfield and collaborators [25]. Briefly, the sonic extract was centrifuged at 27,000 ×g for 20 min, and the resulting cell wall containing pellet was subjected to 2% sodium dodecyl sulfate (SDS) extraction for 2 h at 50°C. The SDS extraction was repeated twice. The protein concentration in the cell wall (CW) fraction was evaluated with Kit 2D Quant (GE Healthcare).

### 2.3. Two-Dimensional-SDS Polyacrylamide Gel Electrophoresis (2D-SDS-PAGE)

For 2D analysis, CW fractions were first desalted performing a gel filtration step (Sephadex G25 column). Proteins were precipitated with cold acetone and resuspended in a reswelling buffer (8 M urea, 2% CHAPS, 0.5% IPG buffer pH 4–7, 20 mM DTT, and 0.004% bromophenol blue). 2D-SDS-PAGE was performed as described by Xolalpa and collaborators [26]. The gels were transferred onto a nitrocellulose membrane (Hybond-ECL GE Healthcare) and the membranes were subjected to Western blot. The sera from 5 positive animals were pooled and diluted to 1 : 100 to detect immunogenic proteins from the CW fraction. The immunoreactive spots were manually excised from a replicate Coomassie blue stained gel and sent to the Mass Spectrometry Center for Biological and Chemical Analysis (CEQUIBIEM) at the School of Exact and Natural Sciences, University of Buenos Aires. The mass spectrometry platform used is made up of UV-MALDI-TOF/TOF Ultraflex II (Bruker Daltonics) and the software Mascot was used to identify proteins from peptide sequence databases. The protein score was calculated as −10∗Log(*P*), where *P* is the probability in which the observed match was a random event. Protein scores greater than 69 are significant (*P* < 0.05).

The proteins identified by MALDI-TOF MS were subjected to bioinformatic analysis including similarity searches with proteins with FN-binding domains. Sequence similarity searches were performed by BlastP (http://blast.ncbi.nlm.nih.gov/Blast.cgi).

### 2.4. Recombinant MAP-EF-Tu: Cloning and Expression Assays

DNA from MAP was purified by the CTAB method as described previously by van Embden and collaborators [27]. PCR amplification was performed to amplify the complete open reading frame of EF-Tu using the forward primer* eftu-fw ggatccgcgaaggcgaagttcgag* (*BamH*I site) and the reverse primer* eftu-rev aagcttctacttgatgatcttgac* (*Hind*III site). The amplified 1,190 bp fragment was cloned into pGEM-T vector (Promega) and directionally subcloned into pRSET-A (Invitrogen). Protein expression was induced with 1 mM isopropyl *β*-D-1-thiogalactopyranoside (IPTG). The protein was expressed as insoluble inclusion bodies and therefore purified using a ProBond Ni-NTA Resin column (Invitrogen) under denaturing conditions with 8 M urea. After purification, EF-Tu was refolded by gradually removing the urea using a refolding buffer (Tris 50 mM pH 8, Arg-HCl, EDTA, GSH 3 mM, GSSG 0.3 mM, and PMSF 1 mM). Protein quantification was performed using BCA protein assay (Pierce) following the manufacturer's recommendations.

### 2.5. Western Blot

Proteins were fractionated on 12% SDS-PAGE, according to Laemmli procedure [28], and then stained with 0.25% Coomassie Brilliant Blue R250 (Sigma) or transferred onto nitrocellulose membranes (Hybond-ECL GE Healthcare). EF-Tu was assayed by Western blotting using 1 : 3,000 dilution anti-His (GE Healthcare) as primary antibody and an alkaline phosphatase-conjugated anti-mouse IgG (Sigma) as secondary antibody (1 : 3,000 dilution). A colorimetric detection was performed using BCIP/NBT (5-bromo-4-chloro-3-indolylphosphate/nitroblue tetrazolium) Color Development (Promega), according to the manufacturer's instructions.

### 2.6. Ligand Blot Assay (LBA)

Five *μ*g of the purified recombinant proteins was electrophoresed in 12% SDS-PAGE gels and transferred onto nitrocellulose membranes (Hybond-ECL, GE Healthcare). Ag85 [15] and AhpC were used as positive and negative controls, respectively. The membranes were blocked with 5% BSA in PBS buffer for 1 h at room temperature and incubated with 20 *μ*g/mL of FN for 24 hs at 4°C. The membranes were then washed three times with PBS and incubated with anti-FN in PBS-BSA 5% (1 : 100) for 2 h at room temperature, followed by a final incubation with anti-Mouse IgG alkaline phosphatase antibody (1 : 30,000) for 1 h at room temperature. The membranes were washed and the colorimetric detection of the bound bait protein was performed.

### 2.7. Dose-Response Curves

The 96-well plates (Polysorp Nunc) were coated with 1 *μ*g of EF-Tu in 200 *μ*L carbonate buffer pH 9.5 at 4°C and incubated overnight. AhpC was included as a negative control. Plates were then blocked and increasing concentrations of FN (0, 1, 10, 20, 50, and 100 *μ*g/mL) were added in a final volume of 200 *μ*L. Protein binding was assessed with hyperimmune anti-FN serum at the dilution of 1 : 100 followed by incubation with HRP-conjugated anti-mouse IgG (Sigma) (1 : 500). Both incubations were performed at 37°C for 1 h. The wells were washed three times, and the colorimetric detection was performed using 280 *μ*L of developing solution 2,2′-azino-bis3-ethylbenzothiazoline-6-sulphonic acid (ABTS) at a concentration of 10 *μ*g/mL (Sigma) and 12 *μ*L of H_2_O_2_ 30% in 10 mL of 0.1 M citrate phosphate buffer at pH 5. The absorbance at 405 nm was determined in a microplate reader Multiskan Spectrum (Thermo Scientific).

### 2.8. Surface Plasmon Resonance (SPR)

Protein-protein interactions were assessed by SPR [29], using a BIAcoreT100 system (GE Healthcare). Briefly, FN was covalently immobilized on the BIAcore carboxymethylated dextran matrix −5 sensor chip (GE Healthcare) according to the manufacturer's instructions. Protein solution of EF-Tu (0, 0.312, 0.625, 1.25, 2.5, 5.0, and 10.0 *μ*M) in 10 mM HEPES, 150 mM NaCl, 3 mM EDTA, 0.005% surfactant P20, pH 7.4 was injected over immobilized FN at a flow rate of 30 *μ*L/min for 1 min at 25°C. The dissociation was carried out with PBS-Tween 0.05% or HBS-P20. Surfaces were regenerated by applying pulses of 10 mM HCl. The KD was determined under equilibrium conditions using a nonlinear BIA evaluation program. The nonspecific binding control consisted of passing the analytes on a free surface that had been previously activated and blocked.

### 2.9. Humoral Response Evaluation by Line Print Immunoassay

The protein EF-Tu and a set of antigens, purified protein derivative of* M. avium* (PPDa), purified protein derivative of* M. bovis* (PPDb), and paratuberculosis protoplasmic antigen (PPA3), were evaluated with different sera. Sera were obtained from 10 healthy animals, from 8 animals with bovine tuberculosis (TBB experimentally infected animals, positive for delayed-type hypersensitivity—DTH—with PPDb and with lesions at the end of the experience), and from 25 PTB naturally infected animals, positive for DTH with PPDa and fecal culture positive. A total of 20 *μ*L of each of the antigens was applied onto a nitrocellulose membrane (Amersham Hybond TM-ECL) using a semiautomatic aerosolizer (Camag Scientific Inc., Wilmington, Delaware) at a concentration of 100 *μ*g/mL. The membranes were blocked and placed in a “mini blotter” (Isogen BioSolutions). This procedure allowed simultaneous analysis of the 45 sera, which were evaluated at dilutions of 1 : 100. After 1 h incubation, sera were aspirated and the membranes were washed. The membranes were then incubated with protein G conjugated to peroxidase (1 : 1,500), washed, and finally developed with chemiluminescence substrate (Pierce ECL Western blotting substrate, Thermo Scientific) according to the manufacturer's directions.

## 3. Results

### 3.1. Analysis of MAP-Cell Wall Proteins

**Figure 1:**
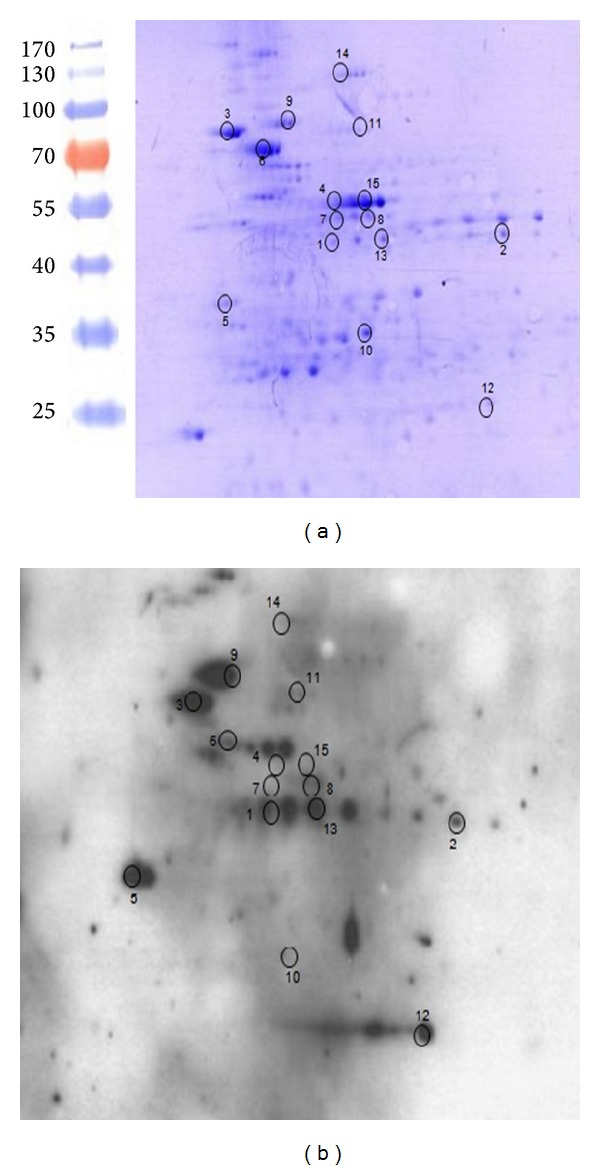
Analysis of MAP-cell wall proteins by 2D-SDS-PAGE. The cell wall protein fraction (CW) of MAP was resolved by 2D-SDS PAGE per duplicate and the resulting gels were (a) stained with Coomassie blue or (b) transferred onto a nitrocellulose membrane and subjected to Western blot. Sera from 5 positive animals were pooled and diluted to 1 : 100 to detect immunogenic proteins from the CW fraction. Molecular weight standards are shown on the left.

As a first screening for surface-exposed immunogenic proteins, the MAP-cell wall (CW) protein fraction was obtained and resolved by 2D-SDS-PAGE and transferred onto a nitrocellulose membrane. Then, the membranes were analyzed by Western blot using a pool of sera from MAP-infected animals. The images were digitalized and visually analyzed (Figure 1). A total of 41 spots corresponding to proteins that were recognized by positive sera were excised from a replicate Coomassie blue stained gel and subsequently identified by MALDI-TOF MS. A total of 18 proteins were identified by this method (Table 1). We focused our interest in proteins that were previously characterized as adhesins in pathogenic microorganisms. Among these proteins, we selected elongation factor Tu (EF-Tu) because the homologous of* Mycoplasma pneumoniae* (identity 65%) and* Acinetobacter baumannii* (identity 72%) functions as a FN-binding protein that facilitates the interactions between bacteria and extracellular matrix [30–32].

The* eftu* gene from MAP was evaluated for orthologous in* M. avium* strain 104 (GenBank accession number NC_008595) and* M. tuberculosis *(Mtb) H37Rv (GenBank accession number NC_000962). In the MAP genome (MAP strain K-10 GenBank accession number NC_002944), this gene was annotated as MAP 4143 and shares 93% of nucleotide identity with that of Mtb and 100% with that of* M. avium*. The identity at the protein level is 100% between MAP and* M. avium* and 97% with its orthologous in Mtb.

We searched for two FN-binding regions (FBR) in the MAP-EF-Tu protein sequence, which has been previously identified in the carboxyl terminus of* Mycoplasma pneumoniae* EF-Tu [32, 33]. The BlastP analysis is shown in Table 2. The comparison of both regions yielded an identity of 73% for FBR1 and 69% for FBR2.

### 3.2. Recombinant Expression and Ligand Blot Assay (LBA)

**Figure 2:**
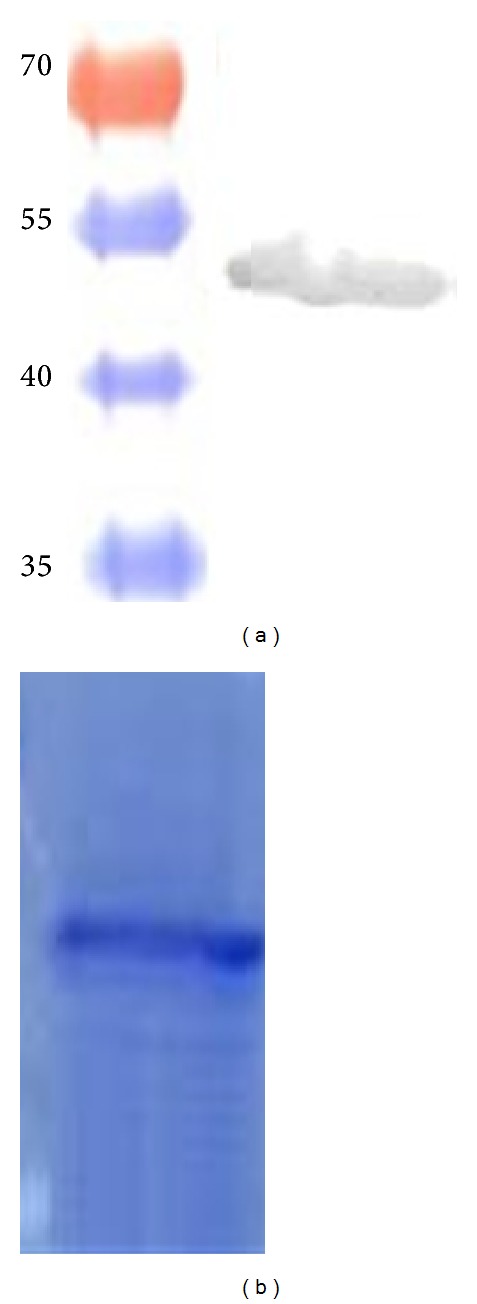
Purified EF-Tu protein using a ProBond Ni-NTA Resin column (Invitrogen). (a) Detection of the protein by Western blot using an anti-Histidine Antibody (Promega). (b) Coomassie blue stained gel showing the purified protein.

**Figure 3:**
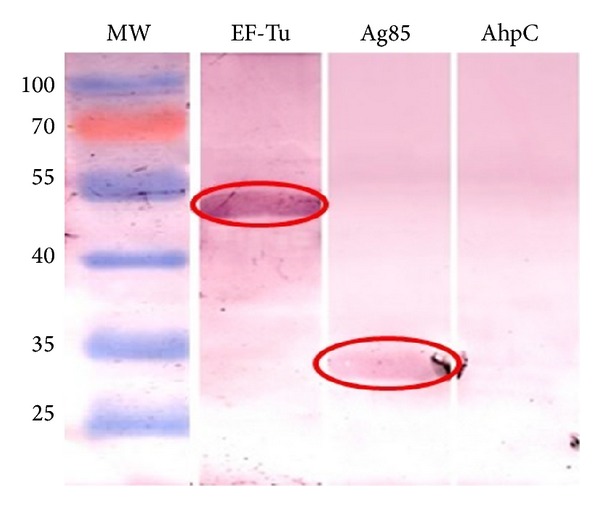
Analysis of FN-binding capability of EF-Tu through a LBA. The blot with the recombinant proteins was incubated with 20 *μ*g/mL FN. Colorimetric detection of the bound bait protein was performed. We observed positive signal indicating the FN-binding capability of EF-Tu and the positive control Ag85 (red circles). AhpC was used as a negative control.

The MAP-EF-Tu protein was heterologously expressed in* E. coli* as a recombinant His-tagged protein and its product was purified under denaturing conditions. Purified EF-Tu protein is showed in Figure 2. The purified protein was used to perform LBA using FN as “bait.” Interactions were detected with anti-FN polyclonal antiserum and an alkaline phosphatase-conjugated antibody with further colorimetric detection of the bound bait. A strong positive signal was observed for EF-Tu and for Ag85 recombinant protein, which was assayed as a positive control. No signal was observed for the negative control, AhpC (Figure 3).

### 3.3. Dose-Response Curves

**Figure 4:**
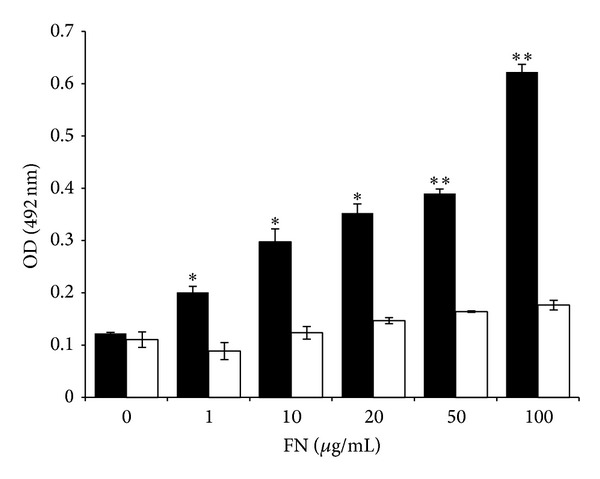
Dose-response curves assayed by ELISA. Plates were coated with EF-Tu (black bars) or AhpC (white bars) used as negative control and incubated with different concentrations of FN. The absorbance, measured at 492 nm, showed a dose-dependent interaction confirming the binding of EF-Tu with FN. Significantly different from values of the control protein AhpC **P < 0.01, *P < 0.05.

We further confirmed FN-EF-Tu interactions using an ELISA assay with some modifications. Plates were coated with 1 *μ*g of EF-Tu or AhpC used as a negative control and then incubated with increasing concentrations of FN. After incubation with the hyperimmune anti-FN serum followed by HRP-conjugated anti-mouse IgG and the corresponding substrate, the absorbance was determined in a microplate reader at 492 nm. We observed a dose-dependent interaction confirming the binding of EF-Tu with FN (Figure 4).

### 3.4. Surface Plasmon Resonance (SPR)

**Figure 5:**
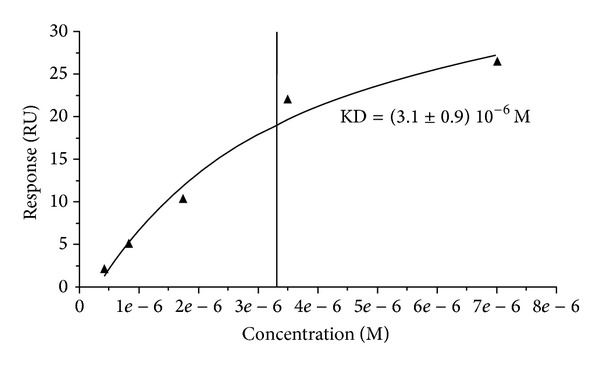
KD determination by surface plasmon resonance. Protein solutions of EF-Tu at different concentrations were injected over immobilized FN. The experiment confirmed FN-EFTu binding, with a KD of (3.1 ± 0.9) 10^−6^ M.

To finally determine the EF-Tu dissociation constant (KD), protein-protein interactions were also assessed through SPR with a BIAcoreT100 system (GE Healthcare). FN was covalently immobilized on the BIAcore CM-5 sensor chip. Solutions of EF-Tu at different concentrations were injected over immobilized FN. The obtained KD was within the *μ*M order, (3.1 ± 0.9) 10^−6^ M (Figure 5). This value is similar to the values of other adhesins previously described [38]. The control using denaturized EF-Tu was not able to bind FN. Therefore, all these experiments confirmed the FN/EF-Tu binding with a moderate affinity through conformational sites.

### 3.5. Humoral Response Evaluation by Line Print Immunoassay

FN-binding proteins could play a role in adhesion to the host and immunomodulation. With this in mind, we analyzed whether EF-Tu is able to stimulate antibody production. Using a line print assay, we evaluated a broader set of sera including TBB-infected animals and negative controls. The sera were obtained from 25 MAP-naturally infected cattle, 8* M. bovis*-experimentally infected cattle, and 10 healthy bovines (negative controls). EF-Tu was recognized by 64% of the MAP positive sera. However, this protein was also recognized by sera of healthy and TBB animals, suggesting the presence of antigenic epitopes conserved among mycobacteria species, including environmental mycobacteria that sensitizes healthy cattle (Table 3).

## 4. Discussion

MAP invades the intestinal tissue primarily through M cells. This could occur through FN-dependent mechanisms that involve the binding of FN to proteins of the MAP CW and to integrin receptors present on the luminal surface of M cells. *β*1 integrins have been identified as the host cell receptors. In addition, the attachment and internalization of MAP by epithelial cells* in vitro* and* in vivo* depend on APA-FN interactions [4]. However, other MAP proteins could be involved in FN binding and they could be important for host-pathogen interactions.

In this study, we have identified immunogenic CW proteins of MAP by 2D and MALDI-TOF MS analysis. From the identified proteins, we selected one candidate based on the similarity with other proteins having the ability to bind ECM molecules in other pathogenic bacteria: elongation factor Tu (EF-Tu).

We first screened its FN-binding capability through a ligand blot assay using FN and anti-FN antibodies. This screening confirmed that, in these conditions, EF-Tu binds FN (Figure 3). Through ELISA assays, using increasing concentrations of FN, we confirmed the interaction in a dose-dependent manner (Figure 4). We finally determine the dissociation constant KD by SPR analysis, which yielded a KD within the order of *μ*M (Figure 5) consistent with previous reports [38].

Several* Mycobacterium* adhesins capable to bind ECM proteins have been identified in mycobacteria, such as antigen 85 complex [11–15], APA [19–23], and GlnA1 [26]. Several reports have demonstrated that these proteins are involved in bacterial dissemination. EF-Tu is a protein responsible for critical steps in protein synthesis [39]. Moreover, this protein is a cytoplasmic protein with unusual CW location among microorganisms. For instance, when* E. coli* is starved for carbohydrates, nitrogen, and phosphate, this protein becomes methylated and associates to the membrane [40]. In addition, this protein was detected as a major CW protein of* Mycobacterium leprae* [41]. On the other hand, EF-Tu has been identified with periplasmic location in* Neisseria gonorrhoeae* [42] and in* E. coli* [43]. In* Lactobacillus johnsonii* and* Listeria monocytogenes*, EF-Tu is also associated to the membrane; in these bacteria this protein mediates binding to mucin [44] and fibrinogen [37], respectively. Recently, it has been reported that EF-Tu binds factor H and plasminogen in* Pseudomonas aeruginosa* [45]. Thus, EF-Tu joins the group of housekeeping enzymes, which includes enolase [46], glyceraldehyde-3-phosphate dehydrogenase [47], and pyruvate dehydrogenase [30], that exhibit unexpected biological functions in addition to their well-defined enzymatic activities. Despite the classification of EF-Tu as a cytosolic protein, Balasubramanian and collaborators [32] have shown that EF-Tu can relocate to the mycoplasma membrane surface with an exposed carboxyl terminus that facilitates FN binding. On the other hand, other reports document the presence of cytosolic proteins, including EF-Tu, on surfaces of different bacteria. Important questions regarding how these proteins translocate to the surface remain unanswered, especially since conventional secretion or anchoring signals are absent. The cytoplasmic enzymefructose-1, 6-bisphosphate aldolase (FBA) has also been found on the surface of several pathogenic bacteria. FBA is a glycolytic enzymethat, despite lacking secretion signals, translocates across the different compartments of the bacterial cell to access the surface, where it binds host molecules and exhibits nonglycolytic functions [40, 41].

The interaction of EF-Tu with the ECM could be a key mechanism during host-pathogen interactions, However, the overall contribution of this adhesin to the host cell binding remains unclear. Although there is a dose-dependent specific binding of EF-Tu to immobilized FN, the affinity of EF-Tu to FN is intermediate. In the present study, EF-Tu displayed a KD in the *μ*M range, similar to other proteins proposed as adhesins. For instance, a surface-exposed protein of leptospira, Lsa20, binds to plasminogen and laminin with a KD in the *μ*M range, postulated as a protein with adherence function and proteolytic activity [38]. Moreover, these adhesins can also bind other proteins of the ECM, such as elastin, laminin, and plasminogen, which contributes to adhesion, to invasion, and to the virulence of the bacteria modulating the immune response [15, 24, 45].

In our study, recombinant EF-Tu was recognized by 64% of sera from MAP-infected cattle, suggesting a putative role in the host immune response. However, sera from noninfected animals also recognize EF-Tu. This result could be due to the presence of EF-Tu in environmental mycobacteria, such as* M. avium*, which shares 100% identity with the MAP protein. Moreover, the suggested role of EF-Tu in the virulence of pathogenic bacteria could be related to the host-pathogen interactions.

Kuo and collaborators [15] demonstrated that reducing the expression of FN on Caco-2 cells that were transfected with FN-siRNA impaired the ability of Ag85 or Ag85-expressing MAP K-10 to bind to the Caco-2 cells. However, MAP K-10 strain binding to FN siRNA-transfected Caco-2 cells showed only a 9.7% reduction (compared with the negative siRNA transfection). This finding suggests that FN is not the only bacterial adhesion ligand contributing to the MAP ability to bind to host cells. Additionally, the MAP Ag85 interaction to FN only partially accounted for MAP's ability to bind host cells. Other surface antigens of MAP K-10 probably participate in additional ECM interactions.

In conclusion, we have identified a novel FN-binding MAP protein, EF-Tu, that could be implicated in the entrance of MAP into the epithelial cells, which is the first step of mycobacteria infection necessary for the progression of PTB.

## Figures and Tables

**Table 1:** Immunogenic CW proteins of MAP identified by MALDI-TOF MS. Proteins were excised from Coomassie blue stained gels and subsequently identified by MALDI-TOF at the Mass Spectrometry Center for Biological and Chemical Analysis (CEQUIBIEM) at the School of Exact and Natural Sciences, University of Buenos Aires. ND: no data.

Spot number	MAP *Locus *	Mtb *Locus *	Gene	Protein function/family	ORF size (bp)	Previously described as envelope protein
1	*MAP1164 *	*Rv1436 *	*gapdh *	Glyceraldehyde-3-phosphate dehydrogenase	1,020	He and de Buck, 2010 (MAP) [34]
2	*MAP1205 *	*Rv1479 *	*moxR *	ATPase	1,143	Mawuenyega et al., 2005 (Mtb) [35]
3	*MAP1325 *	*Rv1630 *	*rpsA S1 *	30S ribosomal protein S1	1,443	Gu et al., 2003 (Mtb) [36]
4	*MAP1367 *	*Rv1658 *	*argG *	Argininosuccinate synthase	1,197	ND
5	*MAP1889c *	*Rv2145c *	*wag31 *	DivIVA family protein	783	He and de Buck, 2010 (MAP) [34]
6	*MAP1962 *	*Rv2220 *	*glnA1 *	Glutamine synthetase A1	1,437	Gu et al., 2003 (MAP) [36]
7	*MAP1998 *	*Rv2245 *	*kasA *	*3-Oxoacyl* synthetase	1,251	He and de Buck, 2010 (MAP) [34]
8	*MAP1999 *	*Rv2246 *	*kasB_1 *	3-Oxoacyl sintase 2 B	1,323	Mawuenyega et al., 2005 (Mtb) [35]
9	*MAP2453c *	*Rv1308 *	*atpA *	ATP synthase subunit alpha	1,665	He and de Buck, 2010 (MAP) [34]
10	*MAP2855c *	*Rv2744c *	*35kd_ag *	Phage shock protein A	828	ND
5	*MAP3152c *	*Rv3075c *	*hpcH/hpaI *	Aldolase/*citrate* lyase	921	Gu et al., 2003 (MAP) [36]
11	*MAP3404 *	*Rv3285 *	*accA3 *	*Carbamoyl-phosphate* synthase subunit A	1,824	Mawuenyega et al., 2005 (Mtb) [35]
12	*MAP3567 *	*Rv0148 *	Hypothetical protein	Short-chain dehydrogenases/reductases	864	He and de Buck, 2010 (MAP) [34]
13	*MAP3651c *	*Rv0215c *	*fadE3_2 *	Acyl-CoA dehydrogenase FadE3	1,218	He and de Buck, 2010 (MAP) [34]
7	*MAP3692c *	*Rv0242c *	*fabG4 *	3-Ketoacyl reductase	1,365	He and de Buck, 2010 (MAP) [34]
14	*MAP3853 *	*Rv0384c *	*clpB *	ATP dependent protease ClpB	2,547	He and de Buck, 2010 (MAP) [34]
3	*MAP3936 *	*Rv0440 *	*groEL2 *	GroEL chaperonin	1,626	He and de Buck, 2010 (MAP) [34]
15	*MAP4143 *	*Rv0685 *	*eftu *	Elongation factor Tu	1,191	He and de Buck, 2010 (MAP) [34]

**Table 2:** BlastP comparison between the two fibronectin binding regions (FBRs) of EF-Tu identified by Balasubramanian and collaborators [37] in *Mycoplasma pneumoniae* (MP) and the homologous regions in MAP.

	MP amino acid region	MAP amino acid region	Identity between MP and MAP (%)
FBR1	192–219	193–220	73
FBR2	340–358	342–360	69

**Table 3:** Reactivity of bovine sera to the protein EF-Tu by line print immunoassay. 20 *μ*L of antigens was applied onto a nitrocellulose membrane and simultaneously confronted to sera from 10 healthy animals, 8 animals with bovine tuberculosis (TBB), and 25 animals with paratuberculosis (PTB). Sixty four percent of MAP positive sera recognized EF-Tu.

Number of sera with positive recognition			
Antigen	Healthy (*n* = 10)	PTB infected (*n* = 25)	TBB infected (*n* = 8)
PPDA	4	16	3
PPA-3	1	18	2
PPDB	6	1	4
EF-Tu	8	16	7
